# Prognostic Factors for Re-Arrest with Shockable Rhythm during Target Temperature Management in Out-Of-Hospital Shockable Cardiac Arrest Patients

**DOI:** 10.3390/jcm8091360

**Published:** 2019-09-01

**Authors:** Seung Mok Ryoo, Dong Hun Lee, Byung Kook Lee, Chun Song Youn, Youn-Jung Kim, Su Jin Kim, Yong Hwan Kim, Won Young Kim

**Affiliations:** 1 Department of Emergency Medicine, University of Ulsan College of Medicine, Asan Medical Center, Seoul 05505, Korea; 2 Department of Emergency Medicine, Chonnam National University Medical School, Gwangju 61469, Korea; 3 Department of Emergency Medicine, College of Medicine, The Catholic University of Korea, Seoul 06591, Korea; 4 Department of Emergency Medicine, College of Medicine, Korea University, Seoul 02841, Korea; 5 Department of Emergency Medicine, Samsung Changwon Hospital, Sungkyunkwan University School of Medicine, Changwon 51353, Korea

**Keywords:** cardiac arrest, ventricular fibrillation, ventricular tachycardia, ventricular premature complex, risk factors

## Abstract

Re-arrest during post-cardiac arrest care after the return of spontaneous circulation is not uncommon. However, little is known about the risk factors associated with re-arrest. A previous study failed to show a benefit of prophylactic antiarrhythmic drug infusion in all kinds of out-of-hospital cardiac arrest (OHCA) survivors. This study evaluated high-risk OHCA survivors who may have re-arrest with shockable rhythm during targeted temperature management (TTM). Medical records of consecutive OHCA survivors treated with TTM at four tertiary referral university hospitals in the Republic of Korea between January 2010 and December 2016 were retrospectively reviewed. Patients who did not have any shockable rhythm during cardiopulmonary resuscitation (CPR) or unknown initial rhythm were excluded. The primary outcome of interest was the recurrence of shockable cardiac arrest during TTM. There were 289 cases of initial shockable arrest rhythm and 132 cases of shockable rhythm during CPR. Of the 421 included patients, 11.4% of patients had a shockable re-arrest during TTM. Survival to discharge and good neurologic outcomes did not differ between non-shockable and shockable re-arrest patients (78.3% vs. 72.9%, *p =* 0.401; 53.1% vs. 54.2% *p =* 0.887). Initial serum magnesium level, ST segment depression or ventricular premature complex (VPC) in initial electrocardiography (ECG), prophylactic amiodarone infusion, and dopamine and norepinephrine infusion during TTM were significantly higher and more frequent in the shockable re-arrest group (all *p* values < 0.05). Normal ST and T wave in initial ECG was common in the non-shockable re-arrest group (*p =* 0.038). However, in multivariate logistic regression analysis, only VPC was an independent prognostic factor for shockable re-arrest (OR 2.806 (95% CI 1.276–6.171), *p =* 0.010). Initial VPC may be a prognostic risk factor for shockable re-arrest in OHCA survivors with shockable rhythm.

## 1. Introduction

While restoration of spontaneous circulation (ROSC) after initial cardiac arrest resuscitation efforts is often successful, some patients subsequently develop circulatory instability and cardiovascular collapse, which manifest as hypotension or cardiac re-arrest by ventricular arrhythmia [1,2]. In Korea, 40.2% of surviving out-of-hospital cardiac arrest (OHCA) patients died during post-cardiac arrest care [3]. The heart is very vulnerable to developing arrhythmia after ROSC. A previous study has suggested that during therapeutic hypothermia, about 40% of patients had Osborn J waves that may be associated with ventricular fibrillation (VF) in patients with structurally normal hearts [4]. Moreover, during post-cardiac arrest care, patients require cardiovascular drugs, which may induce a ventricular arrhythmia. 

Ventricular arrhythmia, which includes the spectrum from ventricular premature complex (VPC) to VF, is associated with ischemic heart disease, particularly in older patients [5]. VPC is especially common and is present in about 50% of all people regardless of heart disease status [6]. Most life-threatening ventricular arrhythmias are VF and pulseless ventricular tachycardia (pVT), which manifest during cardiac arrest [7]. However, previous studies have reported that frequent VPCs also are associated with increased cardiovascular risk and increased mortality even in the general population [8]. Especially since during therapeutic hypothermia the corrected QT segment (QTc) interval is prolonged, VPC may generate VF by the R-on-T phenomenon [4]. 

Re-arrest, defined as the loss of a pulse after sustained ROSC [9], is one of the potential barriers to survival in patients who experience OHCA [10]. Reported incidences of re-arrest vary widely, from 5% to 39% [11,12,13]. In one multicenter registry analysis in the US and Canada 17.5% of ROSC patients experienced re-arrest and re-arrest was inversely associated with survival [14]. Shockable re-arrest including VF and pVT require specific management such as anti-arrhythmic treatment and immediate defibrillation [15]. Therefore bedside preparation of defibrillators as well as prophylactic anti-arrhythmic drug administration may be helpful in patients who have a high risk of shockable re-arrest. 

In the previous studies of re-arrest, the focus has been on prehospital, inpatient, or emergency department settings [9,10,11]. While target temperature management (TTM) after cardiac arrest can provide an opportunity for hypothermia-associated malignant ventricular arrhythmia potential, the incidence of shockable re-arrest during TTM has not been studied. Moreover, there are very limited data on the association between CPR and post-cardiac arrest care variables and the occurrence of shockable re-arrest during TTM.

During TTM, prophylactic antiarrhythmic drug infusion may reduce the incidence of shockable re-arrest. However, a previous propensity-matched study did not identify any benefit of prophylactic amiodarone infusion in all kinds of surviving OHCA patients [16]. We hypothesize that prophylactic anti-arrhythmic drug infusion may be helpful to some OHCA patients who are at risk of shockable re-arrest. The number of shockable re-arrests may be reduced if we can identify patients at high risk of shockable re-arrest who may benefit from prophylactic anti-arrhythmic drug infusion during TTM. The object of this study is to identify risk factors for shockable re-arrest in surviving OHCA patients during TTM.

## 2. Experimental Section

### 2.1. Setting and Study Population

This retrospective, multicentre, observational study was performed at four urban academic emergency departments in the Republic of Korea. Data were extracted from a TTM registry, which prospectively collected data of consecutive patients with non-traumatic comatose OHCA who were treated with TTM between January 2010 and December 2016. The study was approved by the research ethics committees of each hospital. We included consecutive patients who survived from medical-cause OHCA and were treated with TTM. We excluded patients without an initial rhythm record and patients without any documented shockable rhythm. We divided patients by whether they had an experience of re-arrest with shockable rhythm (shockable re-arrest) or did not experience re-arrest or had re-arrest with non-shockable rhythm (non-shockable re-arrest), such as pulseless electrical activity (PEA) or asystole. Re-arrest was defined as loss of pulse following 20 min of sustained ROSC. The grade of VPC was assigned by the ‘Lown grading system for ventricular arrhythmia’ (Table 1) [17].

### 2.2. TTM Protocol

In all comatose OHCA survivors, TTM was induced with intravenous cold saline and cooling devices (Blanketrol II (Cincinnati Subzero Products, Cincinnati, OH, USA), Arctic Sun Energy Transfer Pad (Medivance Corp, Louisville, CO, USA), or COOLGARD3000 Thermal Regulation System (Alsius Corporation, Irvine, CA, USA)). The target temperature of 33 °C was maintained for 24 h and then patients were rewarmed at a rate of 0.25 °C/h to 37.0 °C. Normothermia was then maintained at 36.5–37.0 °C with the same device for 72 h from ROSC. Temperature was monitored with an esophageal temperature probe or rectal temperature probe. Propofol, benzodiazepine, and opioids were used for sedation and analgesia. If required, a neuromuscular blocker was administered to control shivering. All patients received standard intensive care according to institutional protocols.

### 2.3. Data Collection

Demographic and clinical data, including age, sex, previous medical history, initial vital signs, outcomes, and resuscitation profiles, such as cause of arrest, initial rhythm, duration of resuscitation, emergency department (ED) management drugs, and shock, were obtained. Laboratory values on admission were retrieved from the TTM registry. Initial ECG and coronary angiographic findings and vasopressor and inotrope infusion data were obtained from hospital electronic medical records. The primary outcome was the occurrence of repeated cardiac arrest with shockable rhythm during TTM. The secondary outcome was survival to discharge and good neurologic outcome at discharge, which is cerebral performance category 1 or 2 [18].

### 2.4. Statistical Analysis

This study was a secondary analysis of another observational study that compared outcomes of patients who did or did not receive prophylactic amiodarone continuous infusion during TTM. Continuous variables were expressed as means ± standard deviation (SD) or medians with the interquartile range (IQR) if the assumption of a normal distribution was violated. Categorical variables were expressed as numbers and percentages. To analyse baseline characteristics and laboratory values between shockable re-arrest patients and the patients with non-shockable re-arrest, the Student’s *t*-test was used to compare the means of normally distributed continuous variables and the Mann–Whitney U-test was used to compare non-continuous variables. The Chi-squared or Fisher’s exact test was used to compare categorical variables. We evaluated the association between each risk factor and shockable re-arrest during TTM by logistic regression. Risk factors where *p <* 0.1 in the univariate analysis were included in the multivariate analysis with the backward elimination method to find independently associated risk factors. We calculated the odds ratio (OR) with 95% confidential interval (CI) for each model. All tests in this study were two-sided, and a *p*-value < 0.05 was considered to be statistically significant. All statistical analyses were performed using SPSS for Windows version 20.0 (IBM Corp., Armonk, NY, USA). 

## 3. Results

**Figure 1:**
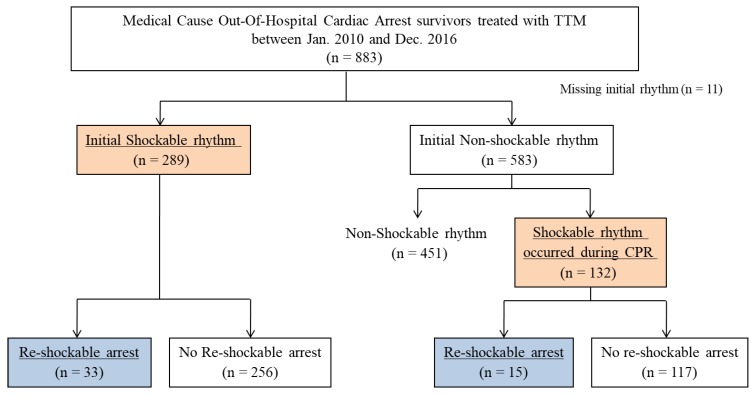
Diagram of the included patients.

Among the 883 medical-cause OHCA survivors treated with TTM, we excluded 11 patients who did not have rhythm data from initial CPR, and 446 patients who did not have initial shockable rhythm and did not have any documented shockable rhythm during CPR. A total of 421 patients were included, 289 with initial shockable rhythm and 132 with shockable rhythm during CPR. Of the included patients, 48 patients (11.4%) developed shockable re-arrest during TTM (Figure 1). 

The median time from ROSC to shockable re-arrest was 6.3 (interquartile range (IQR) 4.0–14.6) h. Re-arrest events occurred during the TTM induction period (54.0%), TTM maintenance (32.0%), rewarming (6.0%), and post-rewarming (8.0%). 

When comparing the shockable re-arrest with non-shockable re-arrest patients, there was no significant difference in mean age (59.0 (4.5–68.0) years vs. 55.0 (45.0–64.0) years, *p =* 0.297) or proportion of males (74.0% vs. 75.1%, *p =* 0.960, respectively). There were no significant differences in past medical history, initial vital signs after ROSC, and initial laboratory findings except serum magnesium levels and the creatinine kinase MB fraction (CK-MB). The initial magnesium and CK-MB were higher in the shockable re-arrest group (2.4 (IQR 2.1–2.8) mg/dL vs. 2.2 (IQR 2.0–2.5) mg/dL, *p =* 0.039; 12.0 (3.3–53.8) ng/mL vs. 7.3 (2.7–26.2) ng/mL, *p =* 0.082, respectively) (Table 2). 

CPR profiles as well as secondary outcomes such as survival discharge and good neurologic outcome were not different between both groups (Table 2). During post-cardiac arrest care, patients with shockable re-arrest had significantly higher levels of ventricular premature complex (VPC), ST segment depression, and lower levels of normal ST and T wave of post-ROSC electrocardiography than patients with non-shockable re-arrest (29.2% vs. 11.5%, *p =* 0.001; 50.0% vs. 35.1%, *p =* 0.044; 10.4% vs. 23.6%, *p =* 0.038, respectively). Of the 57 patients with VPC, there were 27 of grade 1, 13 of grade 2, 7 of grade 3, 5 of grade 4A, 4 of grade 4B, and 1 of grade 5, and their shockable re-arrest rates were 22.2%, 23.1%, 28.6%, 0%, 50.0%, and 100%, respectively. Use of cardiovascular drugs, especially dopamine, norepinephrine, and prophylactic amiodarone infusion, was more frequent in shockable re-arrest patients than in patients with non-shockable re-arrest (79.2% vs. 62.0%, *p =* 0.020; 72.9% vs. 54.2%, *p =* 0.014; 41.7% vs. 26.0%, *p =* 0.023, respectively) (Table 3).

In the multivariate analysis, ST segment elevation, ST segment depression, normal ST and T wave, VPC, initial CK-MB level, prophylactic amiodarone infusion, and cardiovascular drugs during TTM were included. Only VPC in post-ROSC electrocardiography was independently associated with shockable re-arrest (OR 2.806 (95% CI 1.276–6.171), *p =* 0.010) (Table 4). 

## 4. Discussion

In this study we found that VPC in post-ROSC ECG was independently associated with shockable re-arrest during TTM. Patient survival to discharge and good neurologic outcome did not differ significantly between patients with shockable re-arrest or non-shockable re-arrest. 

A recent observational study reported that 24.0% of restored cardiac arrest patients experienced recurrence of cardiac arrest and the median time from ROSC to re-arrest was 5.4 h (IQR 1.1–61.8) [9]. In their subgroup analysis of the 381 patients who experienced re-arrest, 108 (26%) patients had shockable rhythm during initial CPR; 60 (56%) patients had shockable re-arrest, 23 (38%) of whom survived to discharge [9]. Another study with a larger population, found that re-arrest occurred in 17.5% of ROSC patients, 66.4% of whom had a shockable rhythm, and that re-arrest was inversely associated with survival (OR 0.19, 95% CI 0.14–0.26) [14]. In our study the incidence of shockable re-arrest was 11.4%, which was less than the previous study, but the median time of re-arrest was similar (6.3 h (IQR 4.0–14.6)). 

While previous studies have primarily focused on the incidence and outcome of re-arrest, the current study extends these findings by offering risk factors for shockable re-arrest in surviving OHCA patients during TTM. While shockable was not considered, Bhardwaj et al. reported that re-arrest was more associated with black race than white (OR 1.68, 95% CI 1.32–2.13), night time (19:00~07:00) than day time (OR 1.26, 95% CI 1.02–1.57) and more reverse associated in OHCA than in-hospital cardiac arrest (OR 0.46, 95% CI 0.33–0.62) [9]. In our study, VPC was significantly associated with shockable re-arrest by multivariate regression analysis. In Lown’s grading system, the prevalence of grades 1 to 5 were 47.4%, 22.8%, 12.3%, 8.8% (4A), 7.0% (4B), and 1.8% respectively. However, the grade of VPC was not associated with shockable re-arrest. A previous study found that VPC within three months after cardiac arrest was prognostic of subsequent death; however, VPC patterns did not predict recurrent sudden cardiac death [19]. Different from our study, Weaver et al. reported that frequent multiform VPCs, bigeminal or trigeminal and ≥2 repetitive VPCs, were predictors of recurrent sudden cardiac death in OHCA survivors [20]. Because our study did not have enough VPC patients, we could not evaluate the role of frequent multiform VPCs in recurrent sudden cardiac death. In post-myocardial infarction populations, frequent VPC was associated with increased mortality, especially among those with ≥3 consecutive VPCs [21]. Therefore, guidelines recommended catheter ablation or anti-arrhythmia drugs such as amiodarone, beta blockers, and calcium channel blockers for patients with frequent VPCs [7]. 

This study was a first report to predict shockable re-arrest during TTM. Most of the previous studies conducted in prehospital or transporting settings [11,22]. During TTM, especially mild therapeutic hypothermia, one potential complication associated with cooling was electrographic changes including a prolonged PR interval, widening of the QRS complex, increased QT interval, and, sometimes, Osborne waves [23]. In our study we found electrographic changes in VPC were nearly three times higher in patients with shockable re-arrest (OR 2.806 (95% CI 1.276–6.171)). Moreover, post-cardiac arrest patients are often hemodynamically unstable due to the underlying etiology of the arrest, such as myocardial dysfunction and systemic ischemia reperfusion injury, sometimes requiring the administration of vasoactive adrenergic drugs [24]. Unfortunately, many adrenergic drugs increase cardiac arrhythmia [25], especially epinephrine, a non-selective catecholamine with potent alpha and beta adrenergic effects that may increase myocardial workload and disrupt the balance between oxygen supply and demand, increasing the risk of ventricular arrhythmias [26,27,28]. A recent paper reported that more frequent administration of epinephrine during cardiac arrest is associated with the development of secondary ventricular fibrillation or ventricular tachycardia [29]. However, in our study, numbers of epinephrine administration during initial CPR and also continuous infusion during TTM did not differ between patients with shockable re-arrest and non-shockable re-arrest (2.0 (0.0–5.8) vs. 2.0 (0.0–6.0), *p =* 0.410; OR 2.828 (95% CI 0.954–8.377), *p =* 0.061 in multivariate analysis). Moreover, continuous infusion of dopamine and norepinephrine during TTM also were not independently associated (OR 2.172 (95% CI 0.894–5.274), OR 1.304 (95% CI 0.606–2.807), respectively). In this study, amiodarone continuous infusion was more frequently used in patients with shockable re-arrest, likely because physicians use it in more electrically vulnerable patients. However, in the multivariate analysis amiodarone administration was not independently associated with shockable re-arrest. 

This study had several limitations. First of all, it was a prospectively collected retrospective registry based study, so we did not have data associated with cardiac arrhythmia history, such as past history of inherited cardiomyopathy. While no significant associations were identified in the multivariate analysis, some patients received continuous amiodarone infusion, which can suppress shockable ventricular arrhythmia. This study was a secondary analysis of another study that compared continuous amiodarone infusion and not using prophylactic amiodarone for recurrent ventricular arrhythmia during TTM [16]. Therefore, we also only analysed patients who had confirmed shockable rhythm during initial CPR and we had not detail data of patients who had no shockable rhythm despite that they might have a shockable re-arrest. In this study we did not included intoxication and trauma patients because the number of these patients was very small and they rarely experience cardiac arrhythmia. In addition, while some intoxication is very closely associated with arrhythmia, the treatment is different from primary cardiac arrhythmia and most of these patients need sodium bicarbonate; therefore, our findings may not be generalizable.

## 5. Conclusions

In surviving OHCA patients with documented shockable ventricular arrhythmia during initial CPR, VPC in post-ROSC ECG was associated with shockable re-arrest during TTM. While the management of shockable re-arrest remains a challenge, the risk factors of re-arrest identified in this study will be helpful to better understand and address this phenomenon.

## Figures and Tables

**Table 1:** The Lown grading system for ventricular arrhythmia.

Lown Grade	Definition
0	No ventricular premature depolarisations
1	Less than 30 ventricular extrasystoles per hour
2	30 or more ventricular extrasystoles per hour
3	Multiform ventricular extrasystoles
4A	Two consecutive ventricular extrasystoles
4B	Three or more consecutive ventricular extrasystoles
5	R on T (RV/QT less than 1.0)

**Table 2:** Baseline characteristics and cardiopulmonary resuscitation profiles between patients with non-shockable re-arrest and shockable re-arrest.

Characteristics	Total (*n* = 421)	Non-Shockable Re-Arrest (*n* = 373)	Shockable Re-Arrest (*n* = 48)	*p*-Value
Age, years	55.0 (45.0–65.0)	55.0 (45.0–64.0)	59.0 (44.5–68.0)	0.297
Male	333 (75.0)	296 (75.1)	37 (74.0)	0.960
Past medical history				
History of cardiac arrest	7 (1.7)	6 (1.6)	1 (2.1)	0.574
Acute coronary syndrome	78 (18.5)	68 (18.2)	10 (20.8)	0.662
Arrhythmia	27 (6.4)	23 (6.2)	4 (8.3)	0.532
Hypertension	150 (35.6)	131 (35.1)	19 (39.6)	0.543
Diabetes	82 (19.5)	74 (19.8)	8 (16.7)	0.601
Chronic pulmonary disease	10 (2.4)	8 (2.1)	2 (4.2)	0.319
Chronic renal disease	17 (4.0)	16 (4.3)	1 (2.1)	0.706
Liver cirrhosis	3 (0.7)	2 (0.5)	1 (2.1)	0.305
Malignancy	14 (3.3)	14 (3.6)	0 (0.0)	0.385
Vital signs				
Systolic pressure, mmHg	120.0 (93.3–141.8)	120.0 (94.0–142.0)	110.0 (91.0–140.0)	0.247
Diastolic pressure, mmHg	70.5 (60.0–90.0)	72.0 (60.0–90.0)	70.0 (58.0–82.5)	0.546
Pulse rate, beats/min	100 (80.0–120.0)	98.0 (80.0–120.0)	108.5 (81.0–123.0)	0.401
Body temperature, °C	36.1 (35.5–36.4)	36.1 (35.5–36.4)	36.2 (35.5–36.5)	0.651
Laboratory findings, initial				
White blood cell, 103/μL	13.4 (10.6–18.1)	13.1 (10.5–17.9)	14.5 (11.1–21.2)	0.232
Hemoglobin, g/dL	14.2 (12.4–15.4)	14.1 (12.2–15.4)	14.6 (13.1–15.2)	0.421
Sodium, mmol/L	141.0 (138.0–143.0)	141.0 (138.0–143.0)	140.0 (138.0–143.0)	0.273
Potassium, mmol/L	3.8 (3.4–4.3)	3.8 (3.4–4.3)	3.6 (3.2–4.3)	0.158
Calcium, mg/dL	8.0 (7.4–8.8)	8.0 (7.4–8.7)	7.9 (7.4–8.9)	0.960
Magnesium, mg/dL	2.2 (2.0–2.5)	2.2 (2.0–2.5)	2.4 (2.1–2.8)	0.039
Troponin-I, ng/mL	0.591 (0.120–4.485)	0.559 (0.114–4.485)	0.819 (0.141–5.588)	0.256
CK-MB, ng/mL	7.8 (2.7–30.3)	7.3 (2.7–26.2)	12.0 (3.3–53.8)	0.082
BNP, pg/mL	129.6 (41.0–631.0)	131.1 (43.0–631.0)	98.3 (22.4–903.8)	0.392
Witnessed	350 (83.1)	311 (83.4)	39 (81.3)	0.828
Bystander CPR	251 (59.6)	221 (59.2)	30 (62.5)	0.192
Arrest cause				1.000
Presume cardiac cause	386 (91.7)	342 (91.7)	44 (91.7)	
Other medical cause	35 (8.3)	31 (8.3)	4 (8.3)	
Prehospital initial rhythm				0.217
Shockable	289 (68.6)	256 (68.6)	33 (68.8)	
Non-shockable	54 (12.8)	51 (13.7)	3 (6.3)	
Unknown	78 (18.5)	66 (17.7)	12 (25.0)	
Prehospital defibrillation number	1.0 (0.0–2.0)	1.0 (0.0–2.0)	1.0 (0.0–2.0)	0.426
ED defibrillation number	1.0 (0.0–3.0)	1.0 (0.0–3.0)	1.0 (0.0–2.8)	0.637
ED defibrillation energy, Joules	400 (200–1000)	400 (200–1000)	360 (200–690)	0.739
CPR drugs				
Epinephrine	264 (62.7)	229 (61.4)	35 (72.9)	0.213
Vasopressin	15 (3.6)	13 (3.5)	2 (4.2)	0.694
Lidocaine	16 (3.8)	13 (3.5)	3 (6.3)	0.419
Magnesium	28 (6.7)	25 (6.7)	3 (6.3)	1.000
Bicarbonate	58 (13.8)	51 (13.7)	7 (14.6)	0.950
Amiodarone	109 (25.9)	93 (24.9)	16 (33.3)	0.265
ECMO CPR	15 (3.6)	13 (3.5)	2 (4.2)	1.000
No flow time, min	1.0 (0.0–6.0)	1.0 (0.0–6.0)	1.0 (0.0–5.5)	0.835
Low flow time, min	30.0 (20.0–42.0)	29.5 (20.0–42.0)	30.0 (22.0–42.0)	0.335
Survival discharge	327 (77.7)	292 (78.3)	35 (72.9)	0.401
Good neurologic outcome	224 (53.2)	198 (53.1)	26 (54.2)	0.887

Values are expressed as medians (interquartile range), mean ± standard deviation, or numbers (%). Abbreviations: CK-MB, creatinine kinase MB fraction; BNP, B type natriuretic peptide; CPR, cardiopulmonary resuscitation; ED, emergency department; ECMO, extracorporeal membrane oxygenation.

**Table 3:** Cardiovascular management during post-cardiac arrest care between patients with non-shockable re-arrest and shockable re-arrest.

Characteristics	Non-Shockable Re-Arrest (*n* = 373)	Shockable Re-Arrest (*n* = 48)	*p*-Value
Electrocardiography			
ST segment elevation	102 (27.3)	19 (39.6)	0.078
ST segment depression	131 (35.1)	24 (50.0)	0.044
Left bundle branch block	33 (8.8)	3 (6.3)	0.784
Right bundle branch block	40 (10.7)	7 (14.6)	0.424
Normal ST and T wave	88 (23.6)	5 (10.4)	0.038
Prolonged QTc interval	230 (61.7)	32 (66.7)	0.501
Ventricular premature complex	43 (11.5)	14 (29.2)	0.001
Coronary artery angiography			
Interval of ROSC to CAG, hours	4.0 (2.0–101.5)	4.0 (2.0–66.0)	0.548
Left anterior descending stenosis	119 (41.3)	14 (36.8)	0.598
Right coronary artery stenosis	101 (34.5)	9 (23.7)	0.184
Left circumflex artery stenosis	95 (33.0)	8 (21.1)	0.137
Percutaneous coronary intervention	96 (25.7)	13 (27.1)	0.841
Prophylactic amiodarone infusion	97 (26.0)	20 (41.7)	0.023
Cardiovascular drugs during TTM			
Dopamine	230 (62.0)	38 (79.2)	0.020
Norepinephrine	201 (54.2)	35 (72.9)	0.014
Vasopressin	29 (7.8)	4 (8.3)	0.781
Epinephrine	24 (6.5)	7 (14.6)	0.071
Dobutamine	49 (13.2)	9 (18.8)	0.299

Values were expressed as medians (interquartile range), or numbers (%). Abbreviations: VF, ventricular fibrillation; VT, ventricular tachycardia; QTc, corrected QT segment; ROSC, return of spontaneous circulation; CAG, coronary artery angiography; TTM, targeted temperature management.

**Table 4:** Univariate and Multivariate analysis in factors associated with recurrent shockable cardiac arrest.

Risk Factors of Shockable Re-Arrest	Non-Shockable Re-Arrest (*n* = 373)	Shockable Re-Arrest (*n* = 48)	Univariate Analysis OR (95% CI)	*p*-Value	Multivariate Analysis OR (95% CI)	*p*-Value
Electrocardiography						
ST segment elevation	102 (27.3)	19 (39.6)	OR 1.74 (95% CI 0.94–3.24)	0.081	OR 1.278 (95% CI 0.624–2.616)	0.502
ST segment depression	131 (35.1)	24 (50.0)	OR 1.85 (95% CI 1.01–3.38)	0.047	OR 1.320 (95% CI 0.642–2.717)	0.450
Normal ST and T wave	88 (23.6)	5 (10.4)	OR 0.38 (95% CI 0.15–0.98)	0.045	OR 0.513 (95% CI 0.172–1.533)	0.232
Ventricular premature complex	43 (11.5)	14 (29.2)	OR 3.16 (95% CI 1.57–6.36)	0.001	OR 2.806 (95% CI 1.276–6.171)	0.010
Laboratory findings, initial						
CK-MB, ng/mL	7.3 (2.7–26.2)	12.0 (3.3–53.8)	OR 1.00 (95% CI 1.00–1.01)	0.041	OR 1.001 (95% CI 0.999–1.004)	0.389
Magnesium, mg/dL	2.2 (2.0–2.5)	2.2 (2.0–2.5)	OR 1.20 (95% CI 0.82–1.75)	0.344	OR 1.028 (95% CI 0.659–1.605)	0.903
Prophylactic amiodarone infusion	97 (26.0)	20 (41.7)	OR 2.03 (95% CI 1.10–3.77)	0.025	OR 1.533 (95% CI 0.771–3.050)	0.223
Cardiovascular drugs during TTM						
Dopamine	230 (62.0)	38 (79.2)	OR 2.33 (95% CI 1.13–4.82)	0.023	OR 2.172 (95% CI 0.894–5.274)	0.087
Norepinephrine	201 (54.2)	35 (72.9)	OR 2.27 (95% CI 1.17–4.44)	0.016	OR 1.304 (95% CI 0.606–2.807)	0.497
Epinephrine	24 (6.5)	7 (14.6)	OR 2.46 (95% CI 1.00–6.07)	0.050	OR 2.828 (95% CI 0.954–8.377)	0.061

Multivariate analysis was adjusted with ST segment elevation, ST segment depression, normal ST and T wave, ventricular premature complex, initial CK-MB level, prophylactic amiodarone infusion, and cardiovascular drugs during TTM, which had a threshold of *p <* 0.1 in univariate logistic regression analysis. Abbreviations: OR, odds ratio; CI, confidence interval; CK-MB, creatinine kinase MB fraction, TTM, targeted temperature management. Multivariate analysis was done by logistic regression analysis with the entered variables method.
